# Relationship Between Water Fluoridation Rates and Atraumatic Dental Visits to Emergency Departments in the U.S.: An Epidemiological Study

**DOI:** 10.5811/westjem.48503

**Published:** 2025-07-18

**Authors:** Jenna LaColla, Melissa Nelson-Perron

**Affiliations:** * Yale – New Haven Hospital, Department of Emergency Medicine, New Haven, Connecticut; † Touro College of Osteopathic Medicine, Middletown, New York; ‡ Nuvance Health, Department of Emergency Medicine, Poughkeepsie, New York

## Introduction

Dental health is a crucial aspect of overall well-being, and access to safe and effective measures for its maintenance is essential. Water fluoridation, as a public health intervention to prevent dental caries and promote oral health in communities across the United States, has been recognized as an effective strategy since 1945. Numerous studies have demonstrated the positive impact of fluoridation on reducing dental caries and the need for dental treatments; however, there has been ongoing debate about its safety and long-term effects. Although fluoridation is widely acknowledged as an effective public health measure, the connection between water fluoridation and atraumatic dental emergency room visits in the United States remains unknown.

The objective of this study was to compare the incidence of atraumatic dental visits in regions of the United States—Northeast, Midwest, South, and West—with adequate water fluoride content, to those below recommended fluoride thresholds.

## Methods

This is an epidemiological nationwide study. Using the Nationwide Emergency Department Sample (NEDS), we cross-referenced water fluoridation areas in the United States with emergency department visits for atraumatic dental care during 2016–2019. We used the NEDS database and applied weights to extrapolate data to the entire U.S. population, limiting our evaluation to areas where the water fluoride content is available. The CDC State Fluoridation Report served as the database for water fluoridation rates in the Northeast, Midwest, South, and West regions of the U.S. These data were cross-referenced to extrapolate the average incidence of atraumatic dental visits per 100,000 over the noted years, divided by region.

## Results

Analysis of NEDS and the CDC’s State Fluoridation Reports revealed that the Northeast region, with 57.48% fluoridation, had an average incidence of 158.81 atraumatic dental visits per 100,000 from 2016 to 2019. The West, with 58.56% fluoridation, had an average incidence of 101.76 atraumatic dental visits per 100,000. The Southern region, with 79.48% fluoridation, averaged 192.71 visits per 100,000. Lastly, analysis revealed that the Midwest region, with 90.50% fluoridation, averaged 186.40 atraumatic dental visits per 100,000 during the same period. The coefficient of determination (R^2^) for these results is 0.5774.

## Conclusions

Using national data from NEDS and the CDC we were able to analyze incidence rates of atraumatic dental visits compared to fluoridation levels of water in the four regions of the U.S. Our results indicate that there is no clear correlation between water fluoridation and number of visits to the emergency department with atraumatic dental complaints. These findings suggest that while water fluoridation remains an important public health measure, further investigation is needed to understand the multifactorial influences on community dental health outcomes.

